# Mechanical Properties and Thermal Decomposition Mechanism of Glycidyl Azide Polyol Energetic Thermoplastic Elastomer Binder with RDX Composite

**DOI:** 10.3390/polym16182626

**Published:** 2024-09-17

**Authors:** Qili Sun, Xiao-Mei Yang, Guang-Zhong Yin

**Affiliations:** 1 School of Materials Science and Engineering, Nanjing University of Aeronautics and Astronautics, Nanjing 210016, China; 2 Faculty of Design, Innovation and Technology, University of Design, Innovation and Technology (UDIT), Av. Alfonso XIII, 97, 28016 Madrid, Spain; 3 Escuela Politécnica Superior, Universidad Francisco de Vitoria, Ctra. Pozuelo-Majadahonda Km 1.800, 28223 Madrid, Spain; amos.guangzhong@ufv.es

**Keywords:** bonding functional binder, energetic thermoplastic elastomer, polymer composites, mechanical properties, thermal decomposition mechanism

## Abstract

To improve the reinforcement effect between a binder and high solid filler in a propellant formula, grafting the bonding group into the binder to form a neutral polymeric is a practically novel approach to improving the interface properties of the propellant. In this work, a glycidyl azide polyol energetic thermoplastic elastomer binder with a –CN bonding group (GAP–ETPE) was synthesized, and the mechanical and thermal decomposition mechanism of GAP–ETPE with Hexogeon (RDX) model propellants were studied. The stress–strain results indicated that the tensile strength and strain of GAP–ETPE/RDX model propellants were 6.43 MPa and 32.1%, respectively. DMA data showed that the storage modulus (E’) of the GAP–ETPE/RDX model propellants could increase the glass transition temperature (*Tg*) values, those were shifted to higher temperature with the increase in filler RDX percentages. TG/DTG showed the four decomposition stages of the decomposition process of the GAP–ETPE/RDX model propellants, and the thermal decomposition equation was constructed. These efforts provide a novel method to improve GAP–ETPE/RDX propellants mechanical property, and the thermal decomposition behavior of GAP–ETPE/RDX propellants also provided technical support for the study of propellant combustion characteristics.

## 1. Introduction

Solid propellants were applied to medium-sized caliber rockets, and they were mainly composed of binders, high-energy fuels, and other additives, which are divided into thermosetting propellants and thermoplastic propellants. Thermosetting propellants require short-term casting and shaping after adding a curing agent, and they cannot be reused due to their crosslinking structure. In contrast, thermoplastic propellants have the advantages of emergency readiness, repeatable processing, and reuse, which has roused the attention of countries around the world [1,2,3,4]. Beaupre synthesized a glycidyl azide polyol energetic thermoplastic elastomer (GAP–ETPE) binder [5], and found that this binder has the advantages of being more thermally stable and insensitive to mechanical stimuli than expensive crystalline poly (bis(azidomethyl)oxetane (polyBAMO) [6,7]. Thus, the GAP–ETPE binder has attracted many researchers investigating its mechanical properties [8,9,10], rheological properties [11], and thermal safety performance [12], which shows good application prospects for the propellant. Furthermore, scientists often increase the specific impulse of propellants by filling them with high-proportion and high-energy explosive cyclotrimethylene trinitramine (RDX). However, it easily suffers from weak interface properties between GAP–ETPE binder and filled RDX and causes unstable burning properties, even potential explosion and accidents when GAP–ETPE/RDX composite propellant undergoes high thrust during work [13,14].

To improve the interfacial properties of solid composite propellant, Zhang designed and synthesized a thermoplastic binder with bonding function, based on Kim’s idea of a neutral polymeric bonding function binder, which can effectively resolve weak interfacial properties for propellants [15,16]; the results show that the tensile strength of the GAP–ETPE/RDX composite increases with the increase in the number of functional groups.

Regrettably, they did not further investigate the mechanism of interfacial enhanced mechanism. Furthermore, some researchers considered the decomposition process of the GAP–ETPE/RDX; however, they did not study how the filler RDX’s decomposition influences the other component (GAP–ETPE binder). It is necessary to study the thermal decomposition behavior of propellant.

In this study, the GAP–ETPE binder with a –CN-bonding functional group was synthesized, and the interfacial enhanced mechanism between binder and RDX was proposed. Then, the mechanical properties and decomposition behaviors of the GAP–ETPE/RDX propellants were investigated by static mechanics analysis, dynamic mechanical analysis (DMA), TG/DTG, and TG/FTIR.

## 2. Experiments

### 2.1. Materials

**Figure 1:**
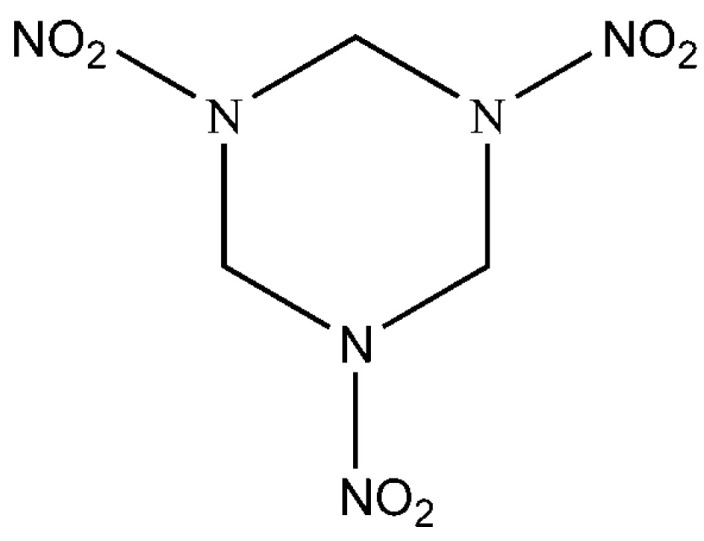
Structures of RDX.

Cyclotrimethylene trinitramine (RDX): Yingguang Chemical Company in Gansu, China, RDX diameter, average particle size of 40 μm; its structures are shown in Figure 1.

**Figure 2:**
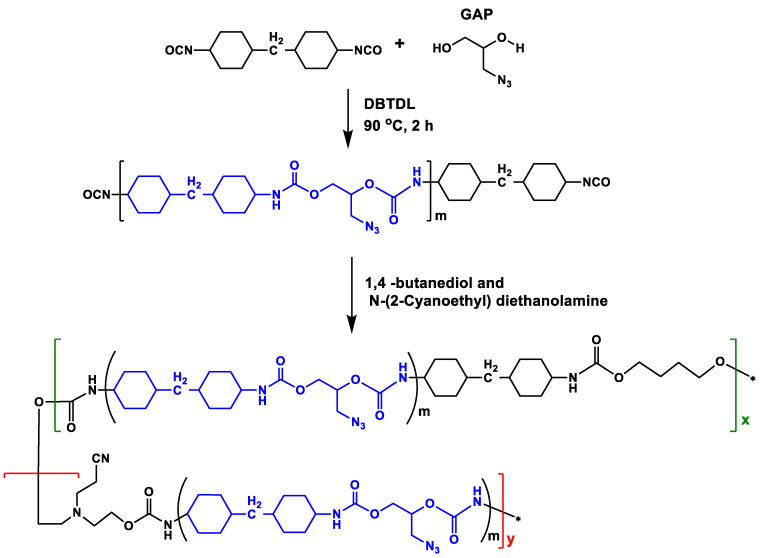
The synthesis process of the GAP–ETPE binder.

The GAP–ETPE binder with the –CN group. The GAP–ETPE with the –CN group was synthesized based on the previous work [8], and the synthesis process was illustrated in Figure 2. The number-average molecular weight (M_n_) was about 30,500 g·mol^−1^, and the polydispersity index (PDI; M_w_/M_n_) of the GAP–ETPEs was 2.06; the ratio of the hard segment (with –CN groups) and soft segment of the GAP–ETPE binder was 3/7.

Preparation of the GAP–ETPE/RDX model propellant [17]: First, the GAP–ETPE was dissolved in tetrahydrofuran to form the GAP–ETPE polymer solution; afterwards, the RDX powder was added to the GAP–ETPE polymer solution at various weight percentages: 10, 30, 50, 70, and 80 and stirred for 0.5 h. The solvent was evaporated and dried in a vacuum oven at 70 °C for 6 h. Finally, the mixture was mixed in an open mill followed vulcanization molding. These samples were named GAP–ETPE/RDX-10, GAP–ETPE/RDX-30, GAP–ETPE/RDX-50, GAP–ETPE/RDX-70, GAP–ETPE/RDX-80, which correspond to RDX filler percentage of 10, 30, 50, 70, and 80, respectively.

### 2.2. Measurements

***Tensile test*:** The stress–strain test was measured using a tensile testing machine (AGS-J, Shimadzu Co., Ltd., Kyoto, Japan) at a constant strain rate of 100 mm/min at room temperature. The dimensions of the dumbbell-shaped specimen samples were in 20 mm × 4 mm × 2 mm (test standards: GB/T 528-1998 [18]).

***Dynamic Mechanical Analyzers* *(DMA)*:** The dynamic thermomechanical properties were measured with a DMA (DMA/SDTA861e, METTLER TOLEDO Co., Ltd., Zurich, Switzerland). All samples were tested in thin film tension mode at an oscillating strain of 5 μm with the frequency of 1 Hz. The experiments were performed at a heating rate of 3 °C/min from −80 °C to 100 °C under a nitrogen atmosphere. The glass transition temperature (*Tg*) was determined as the temperature at the maximum of the tan δ vs. temperature curve.

***Scanning electron microscope* *(SEM):*** Fractured surface morphology of GAP–ETPE/RDX model propellant was studied by Hitachi S4800 (Tungsten source, Hitachi High-Technologies Corp., Tokyo, Japan) Dynamic Mechanical Analyzers scanning electron microscope (SEM) at an accelerating voltage of 15 kV. The fracture surface of all samples was not coated.

***Thermogravimetric Analysis* *(TGA)*:** The decomposition behavior was observed on a METTLER TGA/DSC1/1600 Instrument simultaneous Differential Thermal Analysis (DTA)-TGA((TGA/DSC1/1600, METTLER TOLEDO Co., Ltd., Zurich, Switzerland)). Measurements were usually made with approximately 3 mg of sample using alumina cups. The samples were heated up to 600 °C at a heating rate of 3 °C/min, and a gas flow rate of 40 mL/min of high purity helium was circulated in the cell. Aluminum, silver, and zinc were utilized to calibrate the apparatus. We defined T5% as the onset decomposition temperature of composites, which means 5% weight loss, and *T_max_* as the peak temperature of DTG, which means the highest weight loss rate.

***TGA/FTIR:*** Thermal decomposition mechanism of the GAP–ETPE/RDX was performed by a TG/FTIR. A thermogravimetric analyzer, model TGA/DSC1SF/417-2 (Mettler-Toledo), was used over the range 30–600 °C at a rate of 10 K/min in an argon atmosphere (40 mL/min). A FTIR spectrophotometer, model Nicolet iS10 (Thermo Fisher Scientific, Massachusetts, USA), was linked to the thermogravimetric analyzer to measure the gas products. The FTIR transferring line was heated to 220 °C, and FTIR spectra were recorded from 500 to 4000 cm^−1^ with a resolution of 4 cm^−1^.

## 3. Results and Discussion

### 3.1. Dynamic Mechanical Analysis

**Figure 3:**
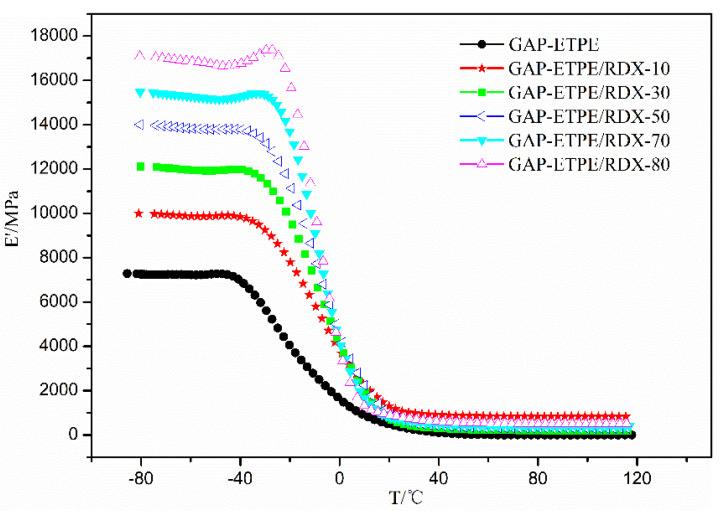
The E’ of samples as a function of temperature.

DMA is selected for characterizing the dynamic mechanical properties of the pure GAP–ETPE and its composites by filling RDX. Figure 3 shows the storage modulus (E’) curves of the pure GAP–ETPE and GAP–ETPE/RDX model propellant with different RDX percentages in the temperature range of −80 °C to 120 °C. It was observed that the E’ value presents a significant enhancement with increasing RDX contents. Typically, the E’ values of pure GAP–ETPE were about 7280 MPa below −40 °C; however, this value increased up to 17,000 MPa after adding RDX to the GAP–ETPE binder (GAP–ETPE/RDX-80). The great improvement in E’ values may be attributed to good matrix–filler interactions because of the induced effect of –CN and –NO_2_; the induced effect model was constructed and will be discussed in Section 3.3.

**Figure 4:**
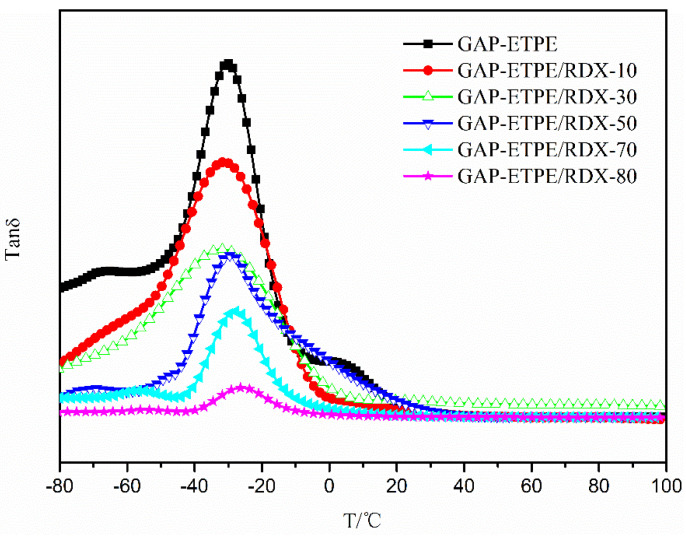
Tan δ of samples as a function of temperature.

The damping factor (*tan δ*) as a function of temperature was shown in Figure 4. The temperature located on peak value can be considered as the glass transition temperature (*Tg*) of GAP–ETPE because of α relaxation corresponding to the glass transition of the polymer composite materials [19], and the related data were summarized in Table 1. From Table 1, it can be seen that the *Tg* of all the GAP–ETPE/RDX model propellants was higher than that of pure GAP–ETPE; meanwhile, the *Tg* value shifted to higher temperature (from −30.1 °C to −26.1 °C) with the RDX percentages increasing, which was attributed to the added RDX particles restricting the mobility of the chain segment of GAP–ETPE [20]. In addition, the pure GAP–ETPE binder showed a narrow *tan δ* peak, while the GAP–ETPE/RDX model propellant clearly showed a broad *tan δ* peak, which indicated that the relaxation process occurred slower than that of the reference sample due to matrix–particle interaction.

### 3.2. Tensile Mechanical Properties Analysis

**Figure 5:**
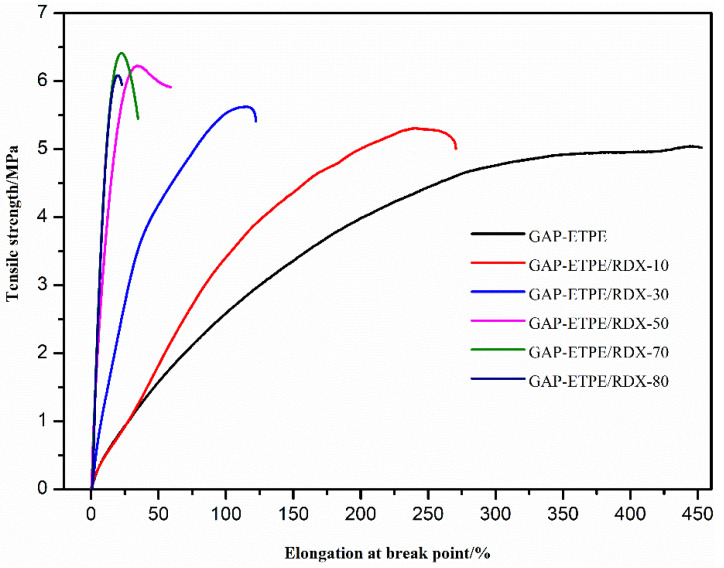
The tensile stress–strain curve of GAP–ETPE/RDX model propellants.

The tensile stress–strain curve of GAP–ETPE/RDX model propellants was shown in Figure 5. It can be observed that the GAP–ETPE/RDX-70 composite propellant exhibited the highest tensile strength. The enhancement of the mechanical properties may be due to good dispersion of GAP–ETPE composite materials filling low percentages RDX in the previous section, as well as good interfacial interaction between RDX and GAP–ETPE with –CN group matrix [21,22]. However, the tensile strength values decreased when the RDX percentages surpassed 70%, which may be associated with inevitable aggregation of the RDX powders at higher filler percentages. Some aggregations can be formed, thus leading to the presence of stress concentrators, and as a consequence, the mechanical properties decrease. On the other hand, the elongation at break point had decreased with the increase in RDX filler, which may be attributed to deformation of GAP–ETPE chains segment and micro-cracks appearing with high RDX percentages. These mechanical data showed that the GAP–ETPE-based propellant with –CN group could be a hopeful application to meet the high performance requirement of the propellant. The typical parameters are listed in Table 2.

**Figure 6:**
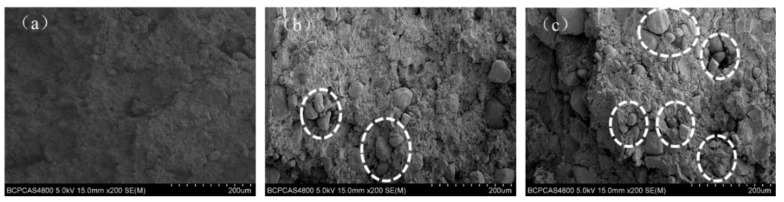
SEM of tensile fracture surface of GAP–ETPE/RDX model propellants with various ratios, 50%, 70% and 80%, corresponding with (**a**–**c**).

In order to confirm the above supposition, the tensile fracture surface morphology of the distribution of RDX in the matrix was examined. Figure 6 shows that the surface micrographs of the GAP–ETPE/RDX model propellants with RDX-50, 70, and 80% correspond to Figure 6a, Figure 6b, and Figure 6c, respectively. As it can be seen from Figure 6, when the filled RDX ratios was less than 70%, there were very few aggregations of RDX grain surface micrographs structures observed on the GAP–ETPE/RDX model propellant. However, as the filler ratios increases to 80%, more aggregations of RDX grain spread on the GAP–ETPE/RDX model propellant surface. These results demonstrate that the aggregations of RDX grain influenced the tensile strength of the GAP–ETPE/RDX model propellant. Typically, the elongation at break decreased, while the tensile strength increased with the increasing of RDX contents.

### 3.3. Interaction-Enhanced Mechanism Analysis

**Figure 7:**
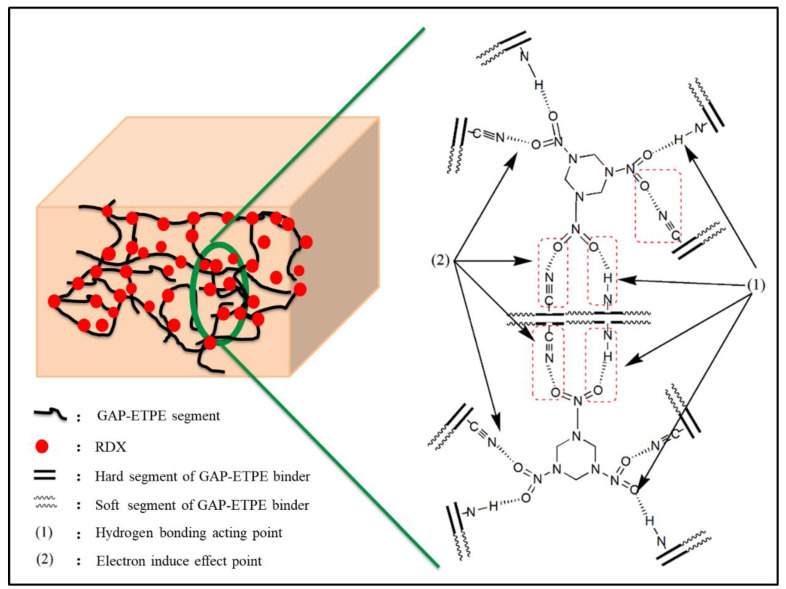
The induced-effect model between –CN and –NO_2_.

In order to understand the reasons for the improved mechanical properties, the molecular structure of the GAP–ETPE and RDX was analyzed, and the electronic interaction model between them was constructed. As shown in Figure 7, the electronegativity of oxygen atom (O) in –NO_2_ group of RDX is well reported to be stronger than the nitrogen atom (N) in the –CN group; O atom exhibits electrophilic property, N atom shows electron donor property, hence, the “induce effect” is generated between –CN and –NO_2_ [5]. When RDX particles were filled into the GAP–ETPE binder containing the –CN group, the physical cross-linking structure was formed, and it produced stronger adhesion force between the GAP–ETPE chains and RDX particles [8]. Furthermore, the filler RDX is a six-membered structure with rigid features, which acted as reinforcement and caused the interactions to restrict the mobility of GAP–ETPE polymer chains with RDX percentages increasing.

### 3.4. Thermal Decomposition Mechanism of GAP–ETPE/RDX

The thermal decomposition of the GAP–ETPE/RDX model propellants is an important factor to predict safety and burning performance [23]. Therefore, it is necessary to understand the thermal decomposition process of pure GAP–ETPE and GAP–ETPE/RDX-50 model propellants.

(1) TG/DTG analysis of GAP–ETPE/RDX

**Figure 8:**
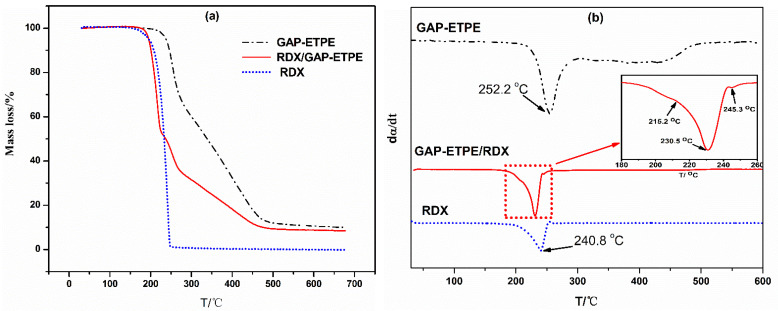
(**a**) TGA and (**b**) DTG curves of samples GAP–ETPE, GAP–ETPE/RDX, and RDX.

Figure 8a,b show the TG results and DTG curves of GAP–ETPE, RDX, and GAP–ETPE/RDX, respectively. In the TGA curve of GAP–ETPE, two characteristic weight loss steps were observed. The first step (a) started at 233 °C, ended at 284 °C, and the maximum mass-losing peak temperature was located at 252 °C, which mainly corresponded to the decomposition of the -N_3_ group. The second stage involved the decomposition of the polyether skeleton of the GAP–ETPE binder between 322 and 478 °C. As for RDX, there was only one loss step, which started at 192 °C and ended at 247 °C, and the maximum mass-loss peak temperature was about 240.8 °C. However, as for GAP–ETPE/RDX, there were four main stages of decomposition, which are clearly depicted in the DTG curve in the Figure 8b inset. We speculated that the first two stages are attributed to RDX decomposition, while the third and fourth are corresponding to the –N_3_ groups and polyether skeleton of the GAP–ETPE binder, respectively.

It can also be seen that the GAP–ETPE/RDX composites have lower degradation temperatures than neat RDX. This decrease is related to the reduced heat loss due to covering RDX by the binder [24]. The mixture of the GAP–ETPE binder presented a small decomposition peak located at about 213.5 °C, and it seemed to present a new phenomenon that the thermal decomposition stage of GAP–ETPE/RDX was divided into two stages between 200 and 240 °C, shown in Figure 8 inset, which have been never reported in the literature. We would like to clarify this phenomenon in the next step.

As expected, the thermal decomposition peak of –N_3_ of GAP–ETPE/RDX is a lower temperature (246 °C) than neat GAP–ETPE. This may be caused by the heat generated from the decomposition of RDX, which acted as a heat source and accordingly caused it to preferentially absorb the heat of GAP–ETPE binder.

**Figure 9:**
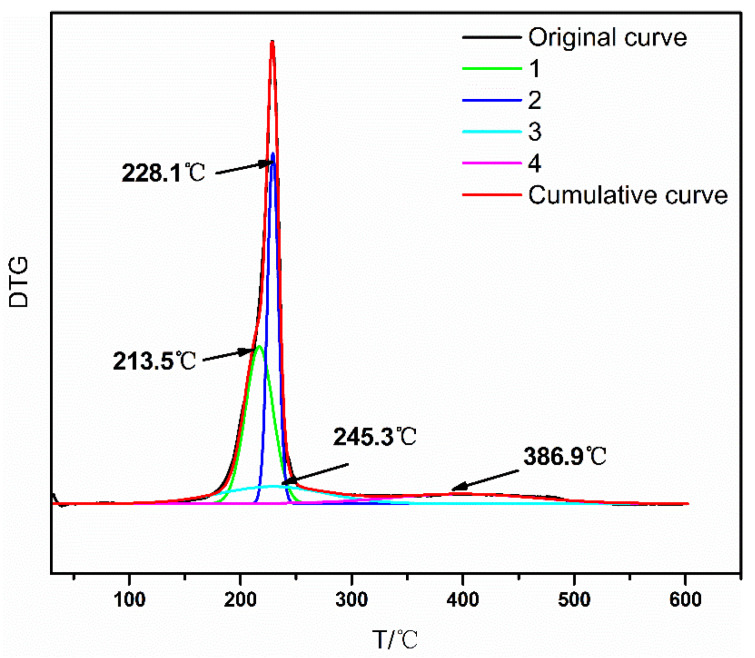
Fitting curve of DTG of GAP–ETPE/RDX.

To understand the thermal decomposition mechanism, the overlapping peaks of the DTG curve were resolved into four stages by Gaussian fitting (Figure 9). By comparing the DTG of GAP–ETPE/RDX, we found that the degradation stage of GAP–ETPE/RDX between 170 and 240 °C corresponded to the decomposition of RDX, and the stage between 200 and 319 °C corresponded to the decomposition of the -N_3_ of the GAP–ETPE binder. The last decomposition stage was related to the polyether and polyurethane skeleton of the GAP–ETPE binder. The peaks of the four stages were at 213.5, 228.1, 245.3, and 386.9 °C, respectively.

(2) TG/FTIR analysis of GAP–ETPE/RDX

**Figure 10:**
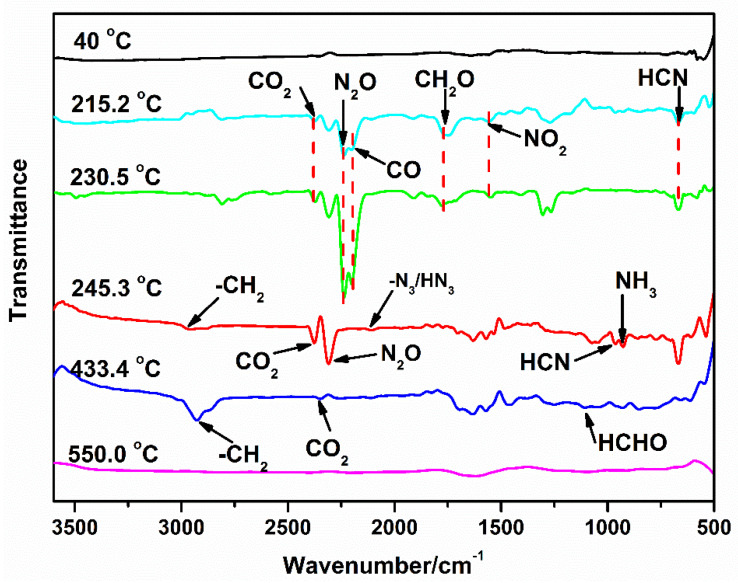
FTIR spectra of gas products during decomposition at respective peaks.

Figure 10 shows the FTIR spectrum of gas products at respective decomposition peaks obtained from Figure 9. In Figure 10, the spectra at 215.2 °C shows the appearance of bands at about 2369, 2238, 2174, 1745, and 713 cm^−1^, which corresponded to the characteristic absorption of CO_2_, N_2_O, CO, CH_2_O, and HCN, respectively; these typical features mainly are attributed to the degradation of the RDX [25]. Thus, we can speculate that the thermal decomposition stage of RDX in GAP–ETPE/RDX is divided into two decomposition stages, which correspond to the slow rate decomposition and self-catalyzed accelerated decomposition stage, respectively. This phenomenon was verified by peak intensity from the TG/FTIR curve in Figure 10.

Furthermore, the gas products containing –CH_2_ (2926 cm^−1^), CO_2_ (2380 cm^−1^), N_2_O (2237 cm^−1^), –N_3_/HN_3_ (2100 cm^−1^), and NH_3_ (964 cm^−1^) release at the temperature of maximum signal (about 245.3 °C) [26]. This is attributed to the degradation of the azide groups, corresponding to the third decomposition stage of the TG test. In addition, at 433.4 °C, the emission of CO_2_, –CH_2_, and HCHO are detected by the appearance of absorption bands at about 2300, 2926, and 1112 cm^−1^, respectively, indicating that the main chain of the GAP–ETPE binder decomposed at this temperature [27]. When the temperature reached 550 °C, all characteristic peaks disappeared, indicating that GAP–ETPE/RDX decomposed completely.

**Figure 11:**
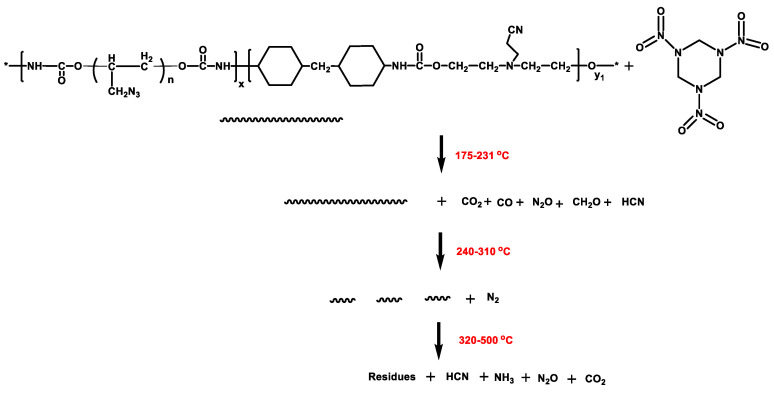
The thermal decomposition process of GAP–ETPE/RDX.

Based on the above analysis, we inferred the decomposition mechanism of GAP–ETPE/RDX, as shown in Figure 11. First of all, at 175–231 °C, the filler RDX thermally decomposed; hydrogen abstraction from RDX occurs through NO_2_, leading to the formation of cis-HONO and a ring intermediate RDX_-H_ with carbon as radical center and subsequently decomposes to methylene nitramine CH_2_NNO_2_, HCN, and NO_2_ [26]. Among of gas products, NO_2_ possessed catalyst effect for further decomposition of RDX [26]. Hence, the process of RDX thermal decomposition was divided into two stages (slow rate decomposition and self-catalyzed accelerated decomposition stage) and released CO_2_, N_2_O, CO, CH_2_O, and HCN from GAP–ETPE/RDX. Secondly, at 240–310 °C, the heat from the RDX decomposition stage transferred into the GAP–ETPE binder, which prompted the –N_3_ group decomposition and caused the maximum decomposition peak temperature ahead time. In this stage, –CH_2_, CO_2_, N_2_O, –N_3_/HN_3_, and NH_3_ was released because of the –N_3_ group decomposition. Finally, at 320–500 °C, the polyether and polyurethane were further decomposed into low molecular weight ether and ketones, and at the same time, N_2_O, HCN, NH_3_, and CO_2_ were released.

## 4. Conclusions

A novel GAP–ETPE binder with bonding functional effect was synthesized, and its composite with RDX was prepared. The mechanical and thermal decomposition performance of GAP–ETPE/RDX composites was investigated, and the following conclusions were obtained:

1. DMA data showed the E’ value of GAP–ETPE/RDX composite increased from 7280 MPa to 17,000 MPa, and the *Tg* value was shifted to higher temperatures, ranging from −30.1 °C to −26.1 °C with the RDX percentages.
2. The static mechanical test showed that GAP–ETPE/RDX composite did not display a de-wetting phenomenon because of the “induce effect” between –CN and –NO_2_ group, which led to a stronger interfacial adhesion force for the GAP–ETPE/RDX model propellant.
3. The TG/FTIR results show that there are four stages during the GAP–ETPE/RDX thermal decomposition: the first two stages are attributed to RDX decomposition process (175–231 °C), which is divided into two stages, one is slow rate decomposition, and the other is self-catalyzed accelerated decomposition stage. The third stage is the azide group (240–310 °C), and the last stage corresponds to the polyether and polyurethane decomposition (320–500 °C).

## Figures and Tables

**Table 1:** The *Tg* value of GAP–ETPE/RDX.

Samples	RDX/%	*Tg*/°C
GAP–ETPE	0	−31.6
GAP–ETPE/RDX-10	10	−30.1
GAP–ETPE/RDX-30	30	−28.6
GAP–ETPE/RDX-50	50	−27.2
GAP–ETPE/RDX-70	70	−26.4
GAP–ETPE/RDX-80	80	−26.1

**Table 2:** Tensile strength, Young’s Modulus, and tensile strain of the samples.

Sample Name	Tensile Strength/MPa	E/MPa	ε_b_/%
GAP–ETPE	5.01 ± 0.52	1.1 ± 0.1	452.3 ± 11.2
GAP–ETPE/RDX-10	5.32 ± 0.73	2.2 ± 0.2	269.2 ± 8.3
GAP–ETPE/RDX-30	5.61 ± 0.51	4.6 ± 0.6	121.5 ± 14.1
GAP–ETPE/RDX-50	6.12 ± 0.32	7.6 ± 0.4	85.9 ± 3.5
GAP–ETPE/RDX-70	6.43 ± 0.65	20.1 ± 0.5	34.7 ± 4.6
GAP–ETPE/RDX-80	6.07 ± 0.58	29.5 ± 2.5	22.5 ± 2.1

## Data Availability

The original contributions presented in the study are included in the article; further inquiries can be directed to the corresponding authors.
